# A Molecular Lateral Flow Assay for SARS-CoV-2 Quantitative Detection

**DOI:** 10.3390/bios12110926

**Published:** 2022-10-26

**Authors:** Panagiotis Maglaras, Ioannis Lilis, Fotini Paliogianni, Vasiliki Bravou, Despina P. Kalogianni

**Affiliations:** 1 Department of Chemistry, University of Patras, 26504 Rio, Patras, Greece; 2 Department of Physiology, Faculty of Medicine, University of Patras, 26504 Rio, Patras, Greece; 3 Department of Microbiology, Medical School, University of Patras, 26504 Rio, Patras, Greece; 4 Department of Anatomy-Histology-Embryology, Medical School, University of Patras, 26504 Rio, Patras, Greece

**Keywords:** COVID-19, coronavirus, rapid test, gold nanoparticles, internal standard, strip

## Abstract

Since the onset of the SARS-CoV-2 pandemic, several COVID-19 detection methods, both commercially available and in the lab, have been developed using different biomolecules as analytes and different detection and sampling methods with high analytical performance. Developing novel COVID-19 detection assays is an exciting research field, as rapid accurate diagnosis is a valuable tool to control the current pandemic, and also because the acquired knowledge can be deployed for facing future infectious outbreaks. We here developed a novel gold-nanoparticle-based nucleic acid lateral flow assay for the rapid, visual, and quantitative detection of SARS-CoV-2. Our method was based on the use of a DNA internal standard (competitor) for quantification and involved RT-PCR, the hybridization of biotinylated PCR products to specific oligonucleotide probes, and detection with a dual lateral flow assay using gold nanoparticles conjugated to an anti-biotin antibody as reporters. The developed test allowed for rapid detection by the naked eye and the simultaneous quantification of SARS-CoV-2 in nasopharyngeal swabs with high specificity, detectability, and repeatability. This novel molecular strip test for COVID-19 detection represents a simple, cost-effective, and accurate rapid test that is very promising to be used as a future diagnostic tool.

## 1. Introduction

Coronavirus disease 2019 (COVID-19), caused by the severe acute respiratory syndrome coronavirus-2 (SARS-CoV-2), has had devastating global health and socioeconomic impacts [1]. The accurate, robust, and early detection of SARS-CoV-2 is critical not only for limiting its spread but also for early diagnosis and proper treatment with newer antiviral drugs [2,3]. Several methodologies that have been discussed in a number of reviews have been developed for SARS-CoV-2 detection involving different analytes, such as viral nucleic acid, viral antigens, antibodies or metabolites, different samples (nasal swabs, nasopharyngeal swabs, saliva, etc.) and different analytical methodologies including RT-PCR, ELISA, lateral flow immunoassays (LFIA), chemiluminescence-based immunoassay (CLIA), loop-mediated isothermal amplification (LAMP), electrochemical assays, CRISPR-mediated technology, etc. [4,5,6]. Among these methodologies, RT-PCR and LFIA are currently used in clinical practice, with advantages and limitations related to sensitivity and specificity, cost, need for trained personnel and advanced instrumentation, assay time, portability and capability to be used for point-of-care testing, and patient self-administration [4,7]. Since the ideal COVID-19 diagnostic test would be a rapid, cost-effective, easy-to-use, efficient, and accurate test, lateral flow assays (LFAs) have attracted great attention, and researchers are currently focusing on improving the sensitivity and specificity of LFA-based SARS-CoV-2 testing, as accuracy is the main limitation for LFA technology [5,8].

Several approaches for enhancing the sensitivity and specificity of lateral flow assays, including pre-amplification steps, signal enhancement with different reporters and readers, and the tailoring of specific and non-specific binding, have been reported for the detection of SARS-CoV-2, with promising analytical metrics [5,9]. For example, Yu et al. (2020) developed a triple fluorescent strip test for the simultaneous detection of three virus-related genes (RdRp, ORF3a, and nucleoprotein (NP)). The total RNA was subjected to RT-PCR. The PCR products labeled with Cy5 fluorescent dye were captured onto the test zones through hybridization to specific immobilized oligonucleotide probes. The detection was completed in 30 min with a detection limit of 10 copies/test [10]. Tetra-primer ARMS-PCR combined with dual-color fluorescent LFA using fluorescent nanobeads was also reported for the simultaneous detection of two SARS-CoV-2 alleles or genes with a detection limit of 2 and 6 copies/μL, respectively [11].

Isothermal amplification combined with LFA has also been introduced for COVID-19 detection. ORF1ab or RdRp and NP genes were simultaneously amplified using RT-LAMP. The amplified products labeled with biotin/fluorescein isothiocyanate (FITC) or fluorescein (FAM)/dixoginenin were detected within 1 h by streptavidin–gold nanoparticles with a detection limit of 12 copies [12] and 20 copies [13], respectively. The same approach was developed for the detection of as low as 3.9 × 10^3^ RNA copies/mL within 15 min [14] or ≥2 copies/μL in <40 min, testing a standard and a NaOH-based protocol for RNA extraction, with comparable results [15]. Finally, a barcoded, isothermal nucleic acid sequence-based amplification (NASBA), combined with neutravidin–carbon nanoparticles, has also been reported (1–2 h, detection limit of <50 RNA copies) [16].

LFAs combined with RT recombinase-aided amplification accomplished analysis in <1 h with a detection limit of 1 copy/μL [17], while a recombinase polymerase amplification (RPA) enabled the detection of 35.4 copies/μL viral cDNA or 0.25–2.5 copies/μL of SARS-CoV-2 plasmid [18]. Finally, RT–RPA combined with DNAzyme provided a detection limit of 10 copies/μL within 1 h [19] or a FAM and biotin-labeled RT–RPA product, 5 copies/μL, detected within 25 min [20]. In another approach, RT and RPA reactions were simultaneously performed for the analysis of 2.8 copies/μL within 15–30 min [21].

Rolling circle amplification (RCA) was also used for isothermal amplification. A padlock probe was hybridized to isolate RNA and, upon ligation, produced a circular template for RCA. The RCA products were hybridized to a labeled detection probe, and after nuclease digestion, the probe was detected using an LFA. The method gave a detection limit of 1.8 × 10^5^ copies/mL within 3 h [22]. Clustered regularly interspaced short palindromic repeats (CRISPRs) mainly in combination with isothermal amplification techniques were also exploited for SARS-CoV-2 detection. Patchsung et al. (2020) used the Cas13a enzyme combined with RT–RPA for the detection of 42 RNA copies [23], while Cao et al. (2022) constructed a home-made portable fluorescent strip reader, providing analysis for 30 min and a detection limit of 0.7 fmol of the spike protein gene and 4.2 fM of the ORF1ab gene [24]. A CRISPR–Cas12-based assay combined with LAMP was also developed for the detection of 10 copies/μL in less than 40 min [25], 10 copies within 1 h [26], and 3–300 copies of RNA within 40–60 min [27], while cross-displacement amplification detected 7 copies within 1 h [28] and RT–RPA, 10 copies in 1 h [29]. CasRx was also exploited for the fluorescent detection of 45 copies/μL [30]. A dual LFA based on CRISPR/Cas9-mediated and RT–RPA was reported for the simultaneous detection of two genes using gold nanoparticles conjugated to specific probes, with a detection limit of 100 RNA copies within 1 h [31]. Finally, a FnCas9 editor-linked uniform detection assay combined with RT-PCR was reported, with a detection limit of 10 RNA copies in 1 h [32].

An amplification-free LFA was also developed using probes specific for three genes and a fluorescent-nanoparticle-labeled monoclonal antibody that binds to double-stranded DNA–RNA hybrids. The method had a total analysis time of < 1 h and a detection limit of 500 copies/mL [33]. Another amplification-free strategy was based on the sandwich hybridization of the RNA target to two specific probes labeled with biotin and FAM, respectively. The complex was then detected by using cysteamine-capped gold nanoparticles for signal enhancement, achieving the detection of 0.02 copies/μL in <30 min [34]. Finally, signal amplification, avoiding RNA isolation and DNA amplification, was achieved through a catalytic hairpin assembly (CHA) reaction detecting 2000 copies/mL in <90 min [35].

We herein developed a novel, dual gold-nanoparticle-based nucleic acid lateral flow assay for the quantitative detection of SARS-CoV-2 based on the use of a DNA internal standard (competitor). The detection is visual by the naked eye, while the densitometric analysis of the two test zones of the strip provides the quantification data and correlation to the initial copies of the target. The method is based on RT-PCR, without RNA isolation, and the detection of the biotinylated PCR products with an LFA using gold nanoparticles conjugated to an anti-biotin antibody as reporters, as well as hybridization to specific oligonucleotide probes, one for SARS-CoV-2 and one for its competitor.

## 2. Materials and Methods

### 2.1. Reagents and Samples

The plasmid that contained the nucleoprotein gene for SARS-CoV-2, the synthetic double-stranded DNA sequence for the internal standard (IS) for SARS-CoV-2 sequence, the synthetic primers, and oligonucleotide probes were purchased from Eurofins Genomics (Εbersberg, Germany) (Table 1). The PrimeScript™ RT reagent Kit was from Takara (Kusatsu, Shiga, Japan), while the polymerase chain reactions (PCRs) were performed using a Kapa2G Fast Ready-Mix Kit (Kapa Biosystems, Basel, Switzerland). Gold nanoparticles were obtained from BBI Solutions (Crumlin, Dublin, Ireland), while polystyrene carboxylated beads with a diameter of 2 μm were purchased from Polysciences Europe GmbH (Hirschberg, Germany). Terminal transferase was from New England Biolabs (Ipswich, MA, USA) and EDC from AppliChem (Maryland Heights, MO, USA). Bovine serum albumin (BSA) was obtained from Serva Electrophoresis GmbH (Duisburg, Germany), and 2-(N-morpholino)ethanesulfonic acid, MES, and anti-biotin antibodies were purchased from Sigma-Aldrich (Saint Louis, MO, USA). The materials used for the fabrication of the strip were previously reported [36].

Nasopharyngeal swab samples were obtained from volunteers that had a negative or positive PCR test. Amplified by real-time PCR products for SARS-CoV-2 testing from nasopharyngeal samples were obtained from the Department of Microbiology, University Hospital, Patras, Greece, which routinely performs the tests for SARS-CoV-2 samples with an established protocol.

### 2.2. Synthesis of Polystyrene Microsphere Conjugates

For the single strip test, polystyrene carboxylated microspheres were coupled to a dT(30) oligonucleotide probe, while for the dual strip test, two different sets of functionalized microspheres were prepared through conjugation to anti-tag oligonucleotide sequences. The first set was compatible with the DNA sequence for SARS-CoV-2, and the second one corresponded to the internal standard DNA sequence. The coupling reaction was performed as described previously [38].

### 2.3. Preparation of Anti-Biotin Antibody-Conjugated Gold Nanoparticles (Antibiotin–AuNPs)

Gold nanoparticles were coupled to anti-biotin antibodies using the protocol of Toubanaki et al. (2009), with few modifications [39]. A volume of 1 mL of the AuNP solution (0.15 pmol/μL) was centrifuged for 20 min at 3.300× *g*, and 500 μL of the supernatant was discarded. The AuNPs were redispersed through vortex-stirring, and the pH of the AuNP solution was adjusted to 9.0 with a 200 mM borax solution. Then, a volume of 460 μL of an anti-biotin antibody that contained 4.6 μg of the antibody in 2 mM borax was gradually added to the AuNP solution. The solution was incubated for 45 min at ambient temperature, in the dark, with frequent stirring. After the incubation step, 100 μL of 100 mg/mL BSA in 20 mM borax was added as a blocking agent, and the solution was incubated for 10 min at room temperature. A 15 min centrifugation step with 4500× *g* was performed afterward, and the supernatant was discarded. The pellet was washed once with 500 μL of 10 g/L BSA in 2 mM borax, followed by centrifugation with 4.500× *g* for 5 min. The obtained anti-biotin–AuNP conjugates were finally reconstituted in 100 µL of 1 g/L BSA and 1 g/LNaN_3_ in 2 mM borax and stored at 4 °C for further use.

### 2.4. Tailing of Complementary Probes with dA

The complementary probes for SARS-CoV-2 and its IS DNA sequences were subjected to a tailing reaction in order to incorporate a poly(dA) tail with terminal transferase (TdT). The reaction had a final volume of 20 μL and contained 1 × TdT buffer, 0.25 mM CoCl_2_, 2 mM dATP, 400 pmol of each of the probes, and 30 U TdT. The reaction took place for 1 h at 37 °C and was stopped by the addition of 2 μL of EDTA 0.5 M, pH 8.0. The tailed with poly(dA) probes were used for the detection and discrimination of the target and its IS through a molecular, single LFA.

### 2.5. Reverse Transcription

At first, the following RT reaction mixture was prepared: For this, 1 μL of dNTP mixture (10 mM each), 1 μL of the reverse primer (1 pmol/μL), 5 μL of the nasopharyngeal sample, and RNase-free ddH_2_O were mixed up to a final volume of 10 μL. The tubes were placed in the thermal cycler for 5 min at 65 °C for denaturation and annealing. Afterward, the following mixture was prepared: Briefly, 10 μL of the reaction mixture from the previous step for each sample, 4 μL of the 5 × PrimeScript Buffer, 0.5 μL of RNase Inhibitor (40 U/μL), 1 μL of PrimeScript RTase, and RNase-free ddH_2_O were mixed up to 20 μL. The tubes were placed in the thermal cycler for 45 min at 42 °C and 5 min at 85 °C. After incubation, the tubes were immediately cooled in an ice bath.

### 2.6. Competitive Quantitative PCR

The DNA sequences for SARS-CoV-2 and its internal standard (IS), i.e., the DNA competitor, were amplified by using a competitive quantitative PCR at a final volume of 20 µL. The PCR reaction pool contained 1 × Kapa 2G Fast Ready Mix, 1 pmol of the forward and 10 pmol of the biotinylated reverse primer, 1 μL of the IS target (10^3^ molecules/μL), and 1 μL of the SARS-CoV-plasmid or cDNA. The conditions of the reaction were, first, a denaturation step at 95 °C for 3 min that was followed by 45 cycles of 95 °C for 5 s, 60 °C for 20 s, and 72 °C for 5 s, and a final extension step at 72 °C for 1 min [37].

### 2.7. Hybridization of the PCR Products to the Complementary Probes

The PCR products were hybridized to the complementary probes, which carried either a poly(dA) tail or a specific tag sequence, at a final volume of 10 μL. Specifically, for the single LFA test, 5 μL of each of the PCR products was mixed with 1 μL of its specific probe, 1 pmol/μL, tailed with poly(dA), 1 μL of NaCl 900 mM, and 3 μL of 1 × PCR buffer. The mixture was then denatured at 95 °C for 2 min and left to hybridize for 10 min at 37 °C.

As for the dual strip test, 5 μL of each of the PCR products was mixed with 1 μL of the tag-probe for the IS DNA sequence and 1 μL of the tag-probe for the SARS-CoV-2 target at an initial concentration of 1 pmol/μL, respectively. Then, 1 μL of NaCl 900 mM and 3 μL of a 1×PCR buffer were added, and the hybridization took place again for 2 min at 95 °C and for 10 min at 37 °C.

### 2.8. Molecular Strip Test—Lateral Flow Assay

A volume of 10 μL of the PCR product that was pre-hybridized to the complementary probe and 5 μL of anti-biotin-functionalized gold nanoparticles were applied onto the conjugate pad of the lateral flow strip. The strip was then immersed into 300 μL of a developing solution that consisted of 1 × PBS pH 7.4, 1% glycerol, 1% Tween, 1% BSA, and 0.5% SDS. The strip was removed from the solution after 10–15 min for the visual detection of the PCR products. Finally, the images of the strips were obtained by using a conventional scanner.

## 3. Results

**Figure 1:**
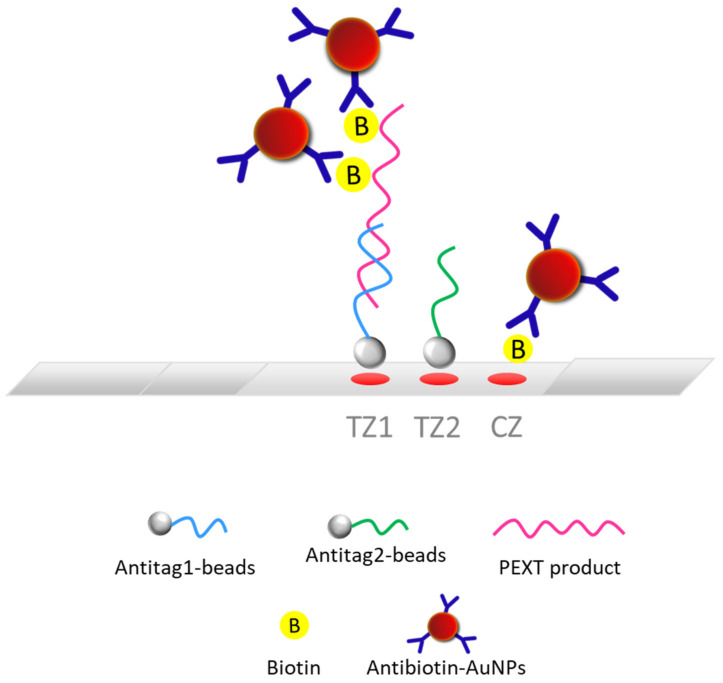
Schematic illustration of the dual molecular lateral flow assay (LFA) with strip test. PEXT: primer extension reaction; AuNPs: gold nanoparticles; TZ: test zone; CZ: control zone.

We developed a molecular lateral flow assay (LFA) with a strip test for the detection and quantification of the SARS-CoV-2 virus. For this purpose, a DNA internal standard (IS), as the DNA competitor, was used for the first time in combination with an LFA to provide the quantitative data. The internal standard used was a double-stranded DNA sequence that was identical to the amplified DNA sequence of the SARS-CoV-2 but differed only in a 24 bp segment (Table 1) that enabled its discrimination from the SARS-CoV-2 DNA sequence with the LFA through a complementary probe. The method was first optimized using a plasmid that contained the DNA sequence of interest for the amplification of the nucleoprotein gene of SARS-CoV-2 via PCR and a double-stranded IS synthetic DNA sequence. Both sequences were amplified via PCR using the same pair of primers. Initially, a single strip for the detection of either the SARS-CoV-2 DNA sequence or the IS was developed. After the optimization of all the steps, a dual molecular strip test was developed for the simultaneous detection of the SARS-CoV-2 target and its IS. For real sample analysis, the method included (i) the collection of nasopharyngeal samples from individuals using the swab and the extraction buffer of a commercially available antigen rapid test; (ii) the nasopharyngeal samples were subjected to reverse transcription (RT) reaction, without RNA isolation, to obtain the cDNA; (iii) the amplification of the SARS-CoV-2 nucleoprotein gene via PCR in the presence of 10^3^ copies of a DNA internal standard; and (iv) the detection of the PCR products with the dual strip test. In detail, after amplification, the PCR products were biotinylated and had a size of 72 bp. The PCR products were then hybridized to specific probes that carried a tag sequence at the 5′ end, complementary to the anti-tag sequences that were attached to the polystyrene beads. The hybrids were then applied to the conjugate pad of the strip along with the pre-deposited gold nanoparticles conjugated with the anti-biotin antibody (anti-biotin–AuNPs). The strip was finally immersed into the developing solution. As the liquid moved upward, a first red spot was formed as the hybrids were captured by the immobilized anti-tag beads for the SRAS-CoV-2 DNA sequence (test zone 1) due to tag/anti-tag hybridization and the accumulation of the anti-biotin–AuNPs through interaction with the biotin of the PCR products. A second red spot was also formed at test zone 2 of the strip as the second set of immobilized anti-tag beads, corresponding to the IS, captured the IS-amplified sequence present in the sample. Finally, a third red spot was generated at the control spot of the strip as the excess of anti-biotin–AuNPs were trapped by the immobilized biotinylated BSA (Figure 1). As for the single strip test, each PCR product was hybridized to each complementary probe that carried a poly(dA) tail at the 3′ end, and the hybrids were then captured onto the test zone of the membrane of the strip through entrapped beads coupled to a poly(dT) oligonucleotide probe.

The images of the dual strips were finally scanned by using a regular scanner, EPSON Perfection V500 PHOTO (Seiko Epson Corporation, Suwa, Japan). Then, the densitometric analysis of the test zones of the strips was performed using the online free image analysis software ImageJ (National Institutes of Health and the Laboratory for Optical and Computational Instrumentation (LOCI, University of Wisconsin, Madison, WI, USA)). The ratio of the signal of the SARS-CoV-2 target to the signal of the IS versus the DNA copies of the SARS-CoV-2 target was used for the quantification of the copies of the virus present in the sample.

### 3.1. Detectability of the Molecular Lateral Flow Assay

To construct a calibration graph, serial dilutions of the SARS-CoV-2 plasmid were prepared in ultrapure water ranging from 1 to 10^8^/μL plasmid copies. Different amounts of the plasmid from 1 to 10^8^ copies were subjected to competitive quantitative PCR in the presence of 10^3^ IS copies. Then, for the single LFA strip test, 5 μL of each of the PCR products was hybridized separately to the complementary probes specific to SARS-CoV-2 and its IS that carried a poly(dA) tail. Finally, 10 μL of each of the hybrids was separately detected with a single strip test. Moreover, for the dual detection, 5 μL of each PCR product was simultaneously hybridized to both specific probes that carried a different tag sequence. The hybrids (10 μL) were then detected with the dual strip test by capturing two different sets of conjugated beads to complementary anti-tag sequences at the two test zones of the strip.

**Figure 2:**
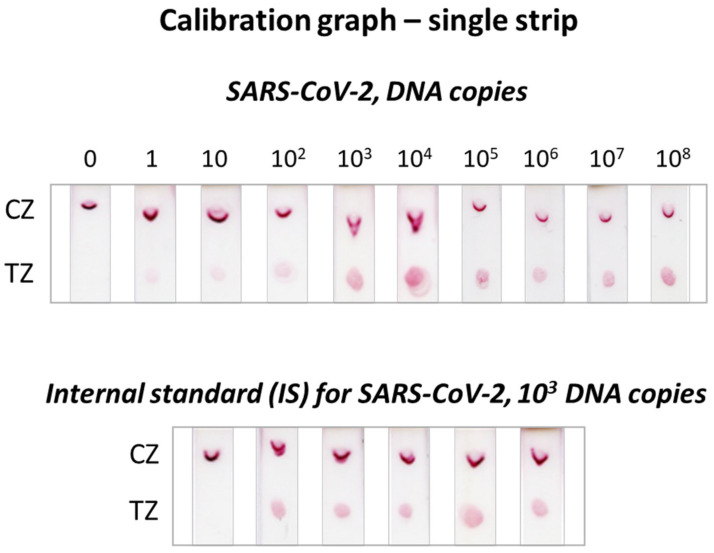
Calibration graph using the single strip test. Different amounts ranging from 1 to 10^8^ plasmid copies for the SARS-CoV-2 DNA sequence were subjected to PCR and detected by using the single strip test, in the presence of 10^3^ DNA copies of the synthetic double-stranded internal standard (IS). TZ: test zone; CZ: control zone.

**Figure 3:**
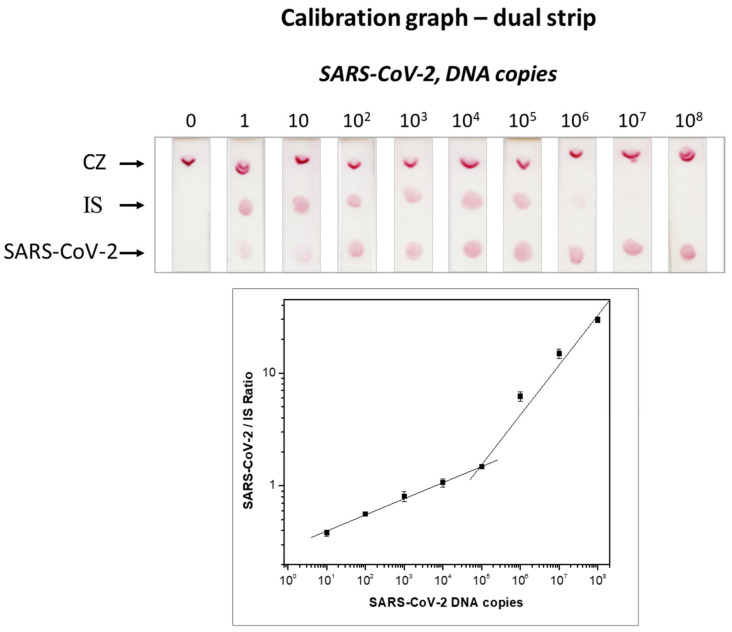
Calibration graph using the dual molecular strip test. Different amounts of plasmid copies for the SARS-CoV-2 DNA sequence, ranging from 1 to 10^8^ copies, were amplified with PCR in the presence of 10^3^ DNA copies of IS. Both sequences were then detected and simultaneously identified with the dual strip test. The ratio of the signal for SARS-CoV-2 over the IS was plotted versus the plasmid copies of SARS-CoV-2. IS: internal standard; CZ: control zone.

The results of the calibration curves using a single strip test are presented in Figure 2, while the calibration curves constructed with the dual strip test are shown in Figure 3. The three replicates of the calibration curve of the dual strip are presented in Figure S1 (Supplementary Materials). The values obtained by the densitometric analysis of the test zones of the strips, along with the mean values and standard deviations for the three replicates, are also presented in Table S1 of the Supplementary Materials. An increase in the signal was observed as the concentration of plasmid DNA increased, while the signal for the IS was almost constant at low concentrations of target DNA and decreased with the increased amount of target DNA. As also observed in Figure 3, a linear correlation of the SARS-CoV-2 target/IS ratio versus the copies of plasmid DNA for SARS-CoV-2 was obtained with the densitometric analysis of the color intensity of both test zones of the dual strip. As low as one copy of synthetic DNA target could be detected after a 40-cycle amplification with PCR.

### 3.2. Clinical Samples

**Figure 4:**
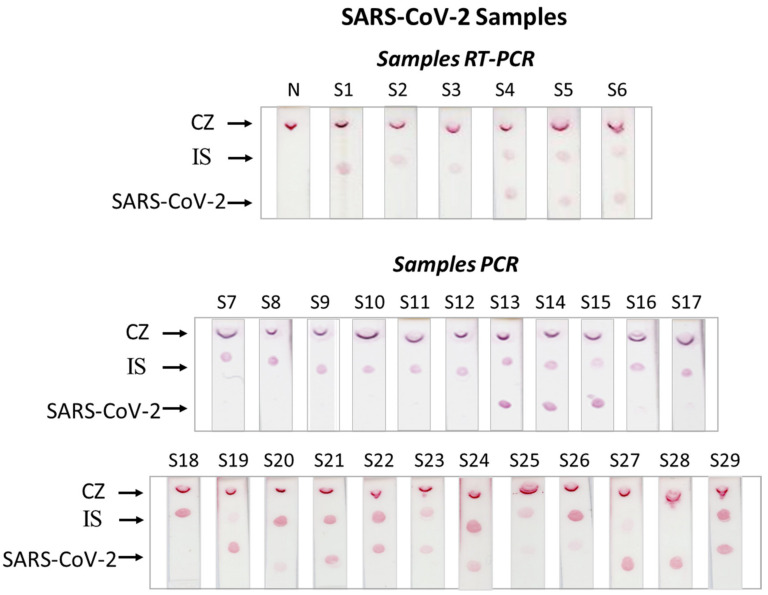
Analysis of nasopharyngeal samples that were subjected to RT-PCR or PCR products that were reamplified using PCR with the dual molecular strip test. IS: internal standard; CZ: control zone.

Two different sets of samples were analyzed by the developed dual strip test. First, the PCR products obtained by real-time PCR from the Department of Microbiology, University Hospital of Patras, Greece, were analyzed using the proposed protocol. These PCR products were diluted 10 times in a 1×PCR buffer, and 1 μL of the diluted sample was subjected to competitive quantitative PCR in the presence of 10^3^ copies of the double-stranded IS DNA sequence. Then, 1 μL of each PCR product was pre-hybridized to the specific probes for SARS-CoV-2 and IS probes and run with the dual strip test. To assess if the method is capable of detecting the SARS-CoV-2 virus in nasopharyngeal samples, three negative samples and three positive samples that were previously characterized by real-time PCR were also analyzed using the proposed method. Briefly, 1 μL of the nasopharyngeal sample that was placed on a swab in the extraction buffer of a commercially available rapid antigen strip test was subjected to reverse transcription (RT). A volume of 1 μL of the RT product was then amplified via PCR, and 1 μL of each PCR product was analyzed with the dual strip test. The analysis of all samples is presented in Figure 4. The results were in concordance with the results obtained with real-time PCR. Samples S1–S3, S7–S12, and S18 were found to be negative for the SARS-CoV-2 virus, while samples S4–S6, S13–S15, and S19–S29 were positive for the virus. Finally, samples S16–S17 were found slightly positive for SARS-CoV-2. Moreover, samples S18–S29 were also quantitatively determined with real-time PCR using an SYBR Green qPCR kit from Kapa Biosystems (Basel, Switzerland), as discussed in the Supplementary Materials, for comparison with the data obtained by the quantitative analysis with the dual strip test. The results are presented in Table S2 (Supplementary Materials). The data obtained by using the strip test were in good agreement with those obtained with real-time PCR.

### 3.3. Specificity of the Molecular Lateral Flow Assay

**Figure 5:**
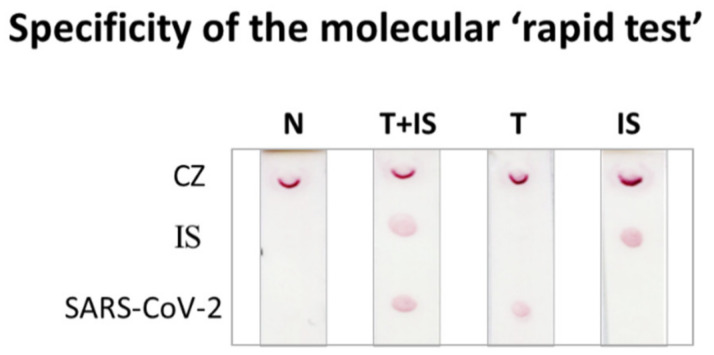
Specificity of the molecular strip test. N: negative; T: target SARS-CoV-2; IS: internal standard; CZ: control zone.

The specificity of the dual strip test was determined. For this purpose, volumes of 5 μL of amplified PCR products for SARS-CoV-2 (10^8^ copies) and IS (10^9^ copies) were separately hybridized to both specific anti-tag probes as previously described. A volume of 5 μL of each PCR product was then analyzed with the dual strip. As observed in Figure 5, a positive result, i.e., a red spot, was identified in the test zone of the strip only for the target and its complementary probe and anti-tag sequence, ensuring the very good specificity of the LFA test. A negative control and a positive control containing both targets were also included in the study.

### 3.4. Repeatability of the Molecular Lateral Flow Assay

**Figure 6:**
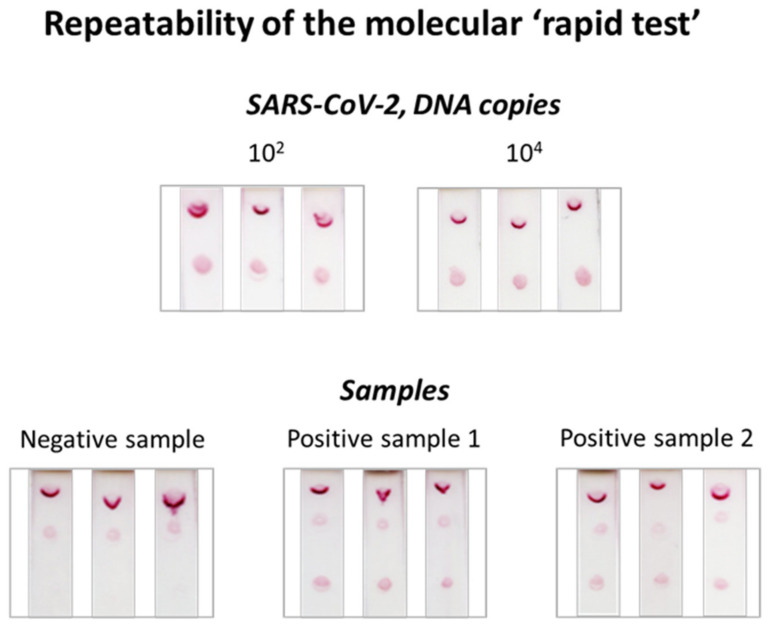
Repeatability of the molecular strip test.

Finally, the repeatability of the molecular strip test was also discerned. Different amounts of plasmid DNA containing 10^2^ and 10^4^ copies, as well as two different positive samples (PCR products) were subjected in triplicate to PCR and analyzed with the dual strip test. The results are shown in Figure 6. The % coefficients of variation (%CVs) were then calculated after densitometric analysis via the ImageJ software of the test zone of the strip that corresponded to SARS-CoV-2. The %CVs were 0.7% and 0.4% for the 10^2^ and 10^4^ plasmid copies and 0.5% and 0.7% for samples 1 and 2, respectively. The very small number of CVs proved the excellent repeatability of the method and the molecular LFA.

## 4. Discussion

A molecular lateral flow assay for SARS-CoV-2 was developed using a methodology that involves a gold-nanoparticle-based nucleic acid lateral flow assay, including a DNA internal standard as the DNA competitor, to allow for simultaneous quantification. The DNA internal standard, i.e., the DNA competitor, had an identical sequence with the amplified sequence of SARS-CoV-2 but differed in a 24 bp central sequence to allow for its discrimination with the LFA. Both sequences shared the same primer pair during simultaneous amplification via PCR and were then hybridized to sequence-specific oligonucleotide probes and detected using a dual strip test. The color intensity of the test zone for SARS-CoV-2 over the test zone of the corresponding DNA competitor was proportional to the target copies present in the sample.

Similar to several previously reported LFA-based platforms for SARS-CoV-2 detection, the developed molecular strip test has most of the advantages of an ideal COVID-19 diagnostic test, such as simplicity, low cost, no need of expensive, special instrumentation, and fast analysis time [5,8,9]. The total analysis time was about 2.5 h, while the analysis with the strip was completed within 10–15 min with the naked eye. It should be noted, however, that compared with other molecular LFA-based tests, the novel incorporation of DNA internal standard in the present assay further provides the advantage of quantification. Moreover, compared with commercially available rapid diagnostic tests, it shows high specificity and sensitivity and also has excellent repeatability with CVs ≤ 0.7% [7,40].

A limitation of the proposed test compared with antigen rapid tests is that it requires an RT-PCR amplification step prior to the strip analysis. However, the integration of nucleic acid amplification strategies such as RT-PCR, isothermal amplification, or CRISPR to LFAs [5,8,9] is necessary to enhance sensitivity, specificity, and detectability, as low diagnostic accuracy is the major hurdle of most of the commercially available point-of-care rapid tests [7,40]. In addition, compared with the conventional RT-PCR techniques and most LFA platforms with integrated amplification technologies, no RNA isolation from nasopharyngeal samples is needed, and a small volume of the sample in the supplied extraction buffer of a commercially available rapid antigen test is directly subjected to reverse transcription reaction that makes the procedure simpler [6,9]. However, amplification-free LFA platforms have been previously reported, as already mentioned, with high performance, but despite their obvious advantages related to the lack of RNA extraction, RT, and amplification steps, they show some limitations regarding, for example, their compromise of signal amplification potential by the limited binding sites and steric hindrance of the antibodies used [33,34].

Finally, further optimization of the test and extensive clinical validation prior to the clinical use is still required, as for most of the recently developed tests, due to the relatively limited sample number for methodological evaluation.

## 5. Conclusions

A novel quantitative molecular lateral flow assay for SARS-CoV-2 in the form of a strip test was developed based on a gold-nanoparticle, nucleic acid lateral flow assay and using a DNA internal standard as the DNA competitor for quantification. The proposed molecular strip test is novel, simple, and cost-effective, with fast analysis time and visual detection, and provides the advantage of simultaneous quantification and shows high detectability, specificity, and repeatability, without the need for expensive or special instrumentation. Even though some limitations exist, the developed molecular strip test shows great promise as a COVID-19 diagnostic tool, also contributing to the accumulating knowledge regarding LFAs for the rapid diagnosis of infectious diseases that could help face future infectious outbreaks. In addition, although this new method needs to be automated to become truly useful in case of epidemics/pandemics, during which it is necessary to analyze a large number of samples in a short time, it still offers the advantage of being cost-effective without the need for special instruments.

## Figures and Tables

**Table 1:** Sequences of the PCR primers, the amplified sequence for SARS-CoV-2, the synthetic IS DNA sequence, the complementary probes, and the anti-tag sequences attached to the polystyrene beads. The PCR primers and the complementary probe for SARS-CoV-2 were previously reported [37].

Name	DNA Sequence (5′ → 3′)
*PCR Primers*	
SARS-CoV-2 Forward	GACCCCAAAATCAGCGAAAT
SARS-CoV-2 Reverse	Biotin-TCTGGTTACTGCCAGTTGAATCTG
*Synthetic Targets*	
SARS-CoV-2 amplified DNA sequence	GACCCCAAAATCAGCGAAATGCACCCCGCATTACGTTTGGTGGACCCTCAGATTCAACTGGCAGTAACCAGA
IS double-stranded	GACCCCAAAATCAGCGAAATGCGTTAGTTAGATTATTGTTAGTTAGCTCAGATTCAACTGGCAGTAACCAGA
*Complementary probes*	
SARS-CoV-2 probe	ACCCCGCATTACGTTTGGTGGACC
IS probe	GTTAGTTAGATTATTGTTAGTTAG
*Complementary tag-probes*	
SARS-CoV-2 tag-probe	**ACGTCACCATCCGAACTTAAAACG**CCGAATTCTCTCACCCCGCATTACGTTTGGTGGACC
IS tag-probe	**AGTCGTAGTCAGAAGTTCAGCAAG**CCGAATTCTCTCGTTAGTTAGATTATTGTTAGTTAG
*Anti-tag sequences*	
anti-tag for SARS-CoV-2	NH_2_-CGTTTTAAGTTCGGATGGTGACGT
anti-tag for IS	NH_2_-CTTGCTGAACTTCTGACTACGACT

## Supplementary Materials

The following supporting information can be downloaded at: https://www.mdpi.com/article/10.3390/bios12110926/s1, Figure S1. Calibration graphs using the dual molecular LFA (n = 3). Different amounts of plasmid copies for SARS-CoV-2 DNA sequence, ranging from 1 to 10^8^ copies, were amplified by PCR in the presence of 10^3^ DNA copies of IS. Both sequences are then detected and simultaneously identified by the dual strip test. The ratio of the signal for SARS-CoV-2 over the IS is plotted versus the plasmid copies of SARS-CoV-2. IS: internal standard; CZ: control zone; Table S1. Contains the densitometric analysis for different concentrations of the target for SARS-CoV-2 and the ratio of the target versus the internal standard (IS), the mean value and the standard deviation (SD) (n = 3). The value of the blank sample has been subtracted from all values; Table S2. Quantitative data of samples obtained by the quantitative molecular dual LFA and real-time PCR (SYBR-Green I).
