# Supplementary material for: A Molecular Lateral Flow Assay for SARS-CoV-2 Quantitative Detection
Source: Biosensors (Basel). 2022 Oct 26;12(11):926. doi: 10.3390/bios12110926 (PMC9687750; doi:10.3390/bios12110926)
Supplement: Supplementary file 1 [file biosensors-12-00926-s001.zip › biosensors-1951735-supplementary.pdf]

biosensors

MDPI

Supplementary Material

A molecular lateral flow assay for SARS-CoV-2 quantitative detection

Panagiotis Maglaras[1], Ioannis Lilis[2,3], Fotini Paliogianni[3], Vasiliki Bravou[4] and Despina P. Kalogianni[1,*]

[1]*Department of Chemistry, University of Patras, 26504 Rio, Greece*

[2]*Department of Physiology, Faculty of Medicine, University of Patras, 26504 Rio, Patras, Greece*

[3]*Department of Microbiology, Medical School, University of Patras, Rio, Patras, Greece*

[4]*Department of Anatomy-Histology-Embryology, Medical School, University of Patras, 26504 Rio, Patras, Greece*

[*]Corresponding author:

Despina P. Kalogianni, Assistant Professor

E-mail address: kalogian@upatras.gr

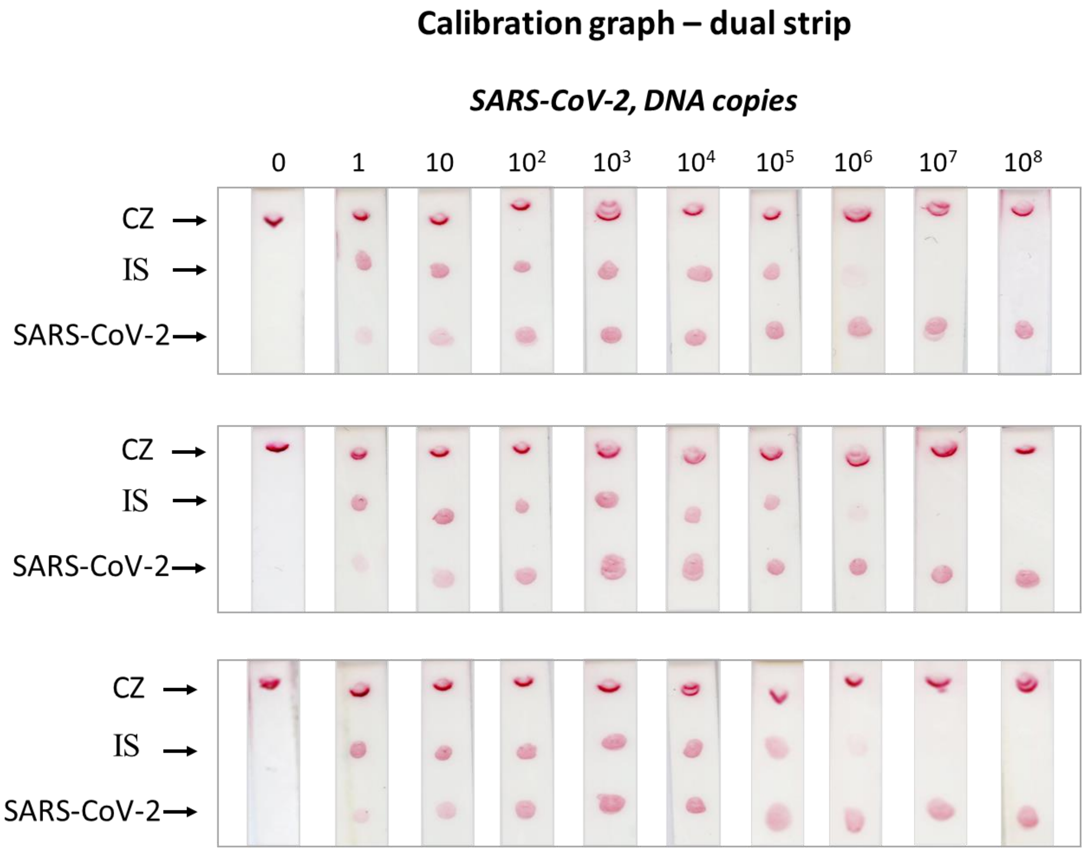

Figure S1. Calibration graphs using the dual molecular LFA (n=3). Different amounts of plasmid copies for SARS-CoV-2 DNA sequence, ranging from 1 to 10^8 copies, were amplified by PCR in the presence of 10^3 DNA copies of IS. Both sequences are then detected and simultaneously identified by the dual strip test. The ratio of the signal for SARS-CoV-2 over the IS is plotted versus the plasmid copies of SARS-CoV-2. IS: internal standard; CZ: control zone.

Table S1. Contains the densitometric analysis for different concentrations of the target for SARS-CoV-2 and the ratio of the target versus the internal standard (IS), the mean value and the standard deviation (SD) (n=3). The value of the blank sample has been subtracted from all values.

Copies	*SARS-CoV-2*			*Mean±SD*	*SARS-CoV-2/ IS*			*Mean±SD*
1	8.3	8.6	11.1	9.3±1.6	0.2	0.2	0.2	0.20±0.02
10	17.5	18.1	19.7	18.4±1.2	0.4	0.4	0.4	0.38±0.04
10^2	32.8	32.6	35.4	33.6±1.6	0.8	0.8	0.8	0.82±0.02
10^3	46.0	44.0	47.2	45.7±1.6	1.1	0.9	1.0	0.99±0.08
10^4	36.8	36.3	48.4	40.5±6.8	1.1	1.3	1.1	1.17±0.09
10^5	41.5	41.1	39.4	40.7±1.1	1.3	1.4	1.3	1.34±0.05
10^6	36.9	44.7	35.8	39.1±4.8	4.8	5.1	3.9	4.6±0.5
10^7	38.0	38.9	43.8	40.2±3.1	38.5	30.8	20.8	30.0±8.8
10^8	49.2	47.1	50.1	48.8±1.6	87.7	65.6	70.9	74.8±11.5

Comparison of quantitative dual strip test with real-time PCR

For comparison with the dual strip test, we performed real-time PCR to the same samples using SYBR-Green I for the detection. For quantification, we also constructed a calibration curve by serial dilutions of the SARS-CoV-2 plasmid and 1-10^8 DNA copies were subjected to PCR, in parallel with the samples. The results from the quantification with the dual LFA and with real-time PCR are presented in Table S2.

Table S2. Quantitative data of samples obtained by the quantitative molecular dual LFA and real-time PCR (SYBR-Green I).

Sample	*DNA Copies*	
	Dual LFA	***Real-time PCR***
S18	negative	negative
S19	1.7×10^3	8.6×10^3
S20	1.1×10^3	5.7×10^3
S21	1.9×10^4	2.5×10^4
S22	52	21
S23	1.8×10^3	6.0×10^3
S24	4.1×10^3	9.7×10^3
S25	<10	<10
S26	<10	<10
S27	$>10^8$	$>10^8$
S28	$>10^8$	$>10^8$
S29	3.4×10^4	7.0×10^4
